# Multimodal RGB–LiDAR Fusion for Robust Drivable Area Segmentation and Mapping

**DOI:** 10.3390/s25185841

**Published:** 2025-09-18

**Authors:** Hyunmin Kim, Minkyung Jun, Hoeryong Jung

**Affiliations:** Department of Mechanical Engineering, Konkuk University, 120 Neungdong-ro, Gwangjin-gu, Seoul 05029, Republic of Korea; sa04101@konkuk.ac.kr (H.K.); minkyung2628@konkuk.ac.kr (M.J.)

**Keywords:** drivable area segmentation, RGB–LiDAR fusion, multimodal perception, real-time mapping

## Abstract

Drivable area detection and segmentation are critical tasks for autonomous mobile robots in complex and dynamic environments. RGB-based methods offer rich semantic information but suffer in unstructured environments and under varying lighting, while LiDAR-based models provide precise spatial measurements but often require high-resolution sensors and are sensitive to sparsity. In addition, most fusion-based systems are constrained by fixed sensor setups and demand retraining when hardware configurations change. This paper presents a real-time, modular RGB–LiDAR fusion framework for robust drivable area recognition and mapping. Our method decouples RGB and LiDAR preprocessing to support sensor-agnostic adaptability without retraining, enabling seamless deployment across diverse platforms. By fusing RGB segmentation with LiDAR ground estimation, we generate high-confidence drivable area point clouds, which are incrementally integrated via SLAM into a global drivable area map. The proposed approach was evaluated on the KITTI dataset in terms of intersection over union (IoU), precision, and frames per second (FPS). Experimental results demonstrate that the proposed framework achieves competitive accuracy and the highest inference speed among compared methods, confirming its suitability for real-time autonomous navigation.

## 1. Introduction

Autonomous mobile robots are increasingly being used in logistics, smart cities, security, and outdoor delivery services. Recently, several countries have allowed mobile robots to operate in pedestrian spaces such as sidewalks and crosswalks, expanding their operation from roads to shared spaces and mixed traffic zones. This expansion implies that the concept of a drivable area for mobile robots is no longer limited to roads. Instead, it refers to any continuous planar surface that the robot is currently navigating. These environments are more diverse and less predictable than structured roads, containing dynamic obstacles such as pedestrians, bicycles, and e-scooters, as well as static obstacles. Changing conditions, such as lighting and weather, add further challenges to perception. To navigate safely in such scenarios, autonomous mobile robots must accurately recognize drivable areas in real time and adapt to variations in their surroundings.

Drivable area recognition methods predominantly rely on RGB images. Lane-detection-based approaches [1,2] effectively extract lanes and boundaries to identify driving paths, but cannot account for dynamic obstacles such as surrounding vehicles or pedestrians. To address this limitation, pixel-level semantic segmentation methods [3] have been introduced, which enable the fine-grained partitioning of road regions and direct recognition of drivable areas. Although such RGB-based segmentation approaches achieve high performance on structured roads such as highways and urban streets, their effectiveness decreases significantly in unstructured environments such as unpaved roads or off-road terrain. In these settings, boundaries are often ambiguous and surface materials are irregular, leading to degraded recognition performance. To mitigate these issues, Zhang et al. [4] proposed segmentation-based recognition in complex unstructured areas such as parks, and Lee et al. [5] attempted drivable area detection on forest roads. However, their methods still suffered performance degradation in multipath terrains and required additional training under varying illumination conditions. Therefore, despite leveraging rich visual cues, RGB-based drivable area recognition has fundamental limitations, including insufficient handling of dynamic obstacles, degraded performance in unstructured environments, and vulnerability to illumination changes and shadows.

To overcome these challenges, LiDAR-based approaches have also been widely studied and applied because of their robustness against illumination changes, ability to capture precise spatial geometry, and effectiveness at obstacle detection. Asvadi et al. [6] proposed a method for detecting both static and moving obstacles in driving environments, whereas Xie et al. [7] developed a technique for identifying negative obstacles such as ditches and depressions, effectively excluding non-drivable areas. Steinke et al. [8] introduced GroundGrid, a LiDAR-based method for ground segmentation and terrain estimation, which provides a reliable foundation for drivable area recognition. However, this approach primarily focuses on separating the ground from non-ground regions and lacks a semantic understanding of surface-level features such as lane markings, curb boundaries, or pavement patterns. To overcome this limitation, Ali et al. [9] proposed a 3D-LiDAR-based road segmentation framework that exploits angular road structures to identify drivable areas in deteriorating road regions. LiDAR-based methods have inherent limitations, as they struggle with low-reflectivity or irregularly shaped objects, and low-channel sensors often produce sparse point clouds that hinder accurate geometric reconstruction.

Several large-scale semantic datasets have been developed to overcome these limitations and support robust LiDAR-based learning. SemanticKITTI [10] offers densely annotated LiDAR sequences collected across various driving scenes, enabling detailed semantic learning. Leveraging such resources, Cylinder3D [11] adopts a cylindrical projection strategy that improves segmentation performance under sparse point cloud conditions. Other datasets [12,13,14] and models [15,16,17,18,19,20] have also emerged, continuously advancing 3D segmentation capabilities in complex outdoor environments.

Considering the limitations of relying solely on LiDAR data, recent studies have explored multimodal approaches integrating RGB imagery with LiDAR data. By leveraging the complementary strengths of visual and spatial cues, these methods have achieved robust performance under varying illumination, dynamic obstacles, and complex scene conditions [20]. Xue et al. [21] addressed the challenge of drivable area detection on dark, unstructured roads by combining CNN-based fusion with surface normal estimation. Candan et al. [22] applied a U-Net-based fusion framework integrating RGB images with LiDAR range representations, thereby improving drivable area recognition in structured road environments. Despite these advancements, performance still depends heavily on sensor configurations such as the LiDAR channel count and resolution.

Most RGB–LiDAR fusion models, as well as LiDAR-based learning models, are designed and developed under fixed sensor setups, assuming consistent resolution and channel count. When these parameters change, most existing methods require retraining for deployment in new environments [23]. This retraining requirement creates a critical dependency. It becomes especially problematic in real-world scenarios where sensor configurations vary widely, such as in autonomous delivery robots, mobile platforms, or other systems deployed across diverse environments. Therefore, there is a growing need for a flexible and robust perception framework that can operate consistently across varying sensor configurations without additional model tuning. This need is especially critical for systems that demand performance and scalable deployment.

To overcome these challenges, this study proposes a practical framework for drivable area perception and mapping, designed to be both sensor-agnostic and modular in architecture. The system separates RGB and LiDAR preprocessing prior to fusion. This design allows seamless adaptation to various sensor configurations without retraining. In addition, it achieves high computational efficiency, suitable for real-time robotic navigation while maintaining recognition performance comparable to learning-based methods. The key contributions of this study are summarized as follows:

1. Sensor-agnostic local drivable area recognition

We present a robust drivable area recognition method based on modular RGB–LiDAR fusion. The system processes RGB segmentation and LiDAR-based ground estimation independently, enabling reliable performance across various sensor types and resolutions. A LiDAR scaling technique is introduced to address point cloud sparsity and enhance the geometric continuity of detected drivable surfaces. Additionally, the perception pipeline is fully modular. Each component can be adapted or replaced independently, according to the available hardware. This flexibility supports seamless deployment across diverse robotic platforms and improves both scalability and integration efficiency.

2. Real-time global drivable area mapping

We propose a method for generating a global drivable area map in real time. The local drivable area identified in each frame is transformed into a global coordinate frame through SLAM-based localization. The system incrementally integrates local recognition results over time. This process constructs a global drivable area map that captures both the surrounding environment and accumulated perception. The resulting map enables stable and reliable path planning in dynamic environments.

## 2. Methods

### 2.1. System Overview

**Figure 1:**
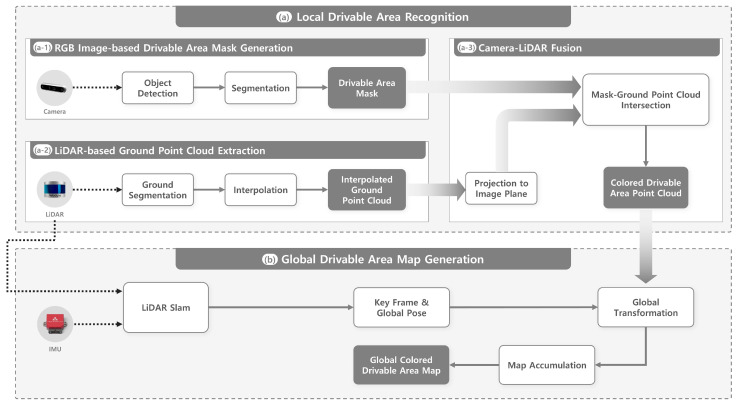
System overview of the proposed drivable area recognition and map generation system. (**a**) Local drivable area recognition: (a-1) generate drivable area masks for road and sidewalk using a “you only look once” (YOLO)-based segmentation model, (a-2) extract ground point clouds from LiDAR data and apply interpolation to mitigate point sparsity, and (a-3) fuse camera and LiDAR data by projecting the ground point cloud onto the image and keeping only points within the drivable area mask. (**b**) Global drivable area map generation: transform the drivable area point cloud into the global coordinate frame using SLAM and accumulate it.

**Figure 2:**
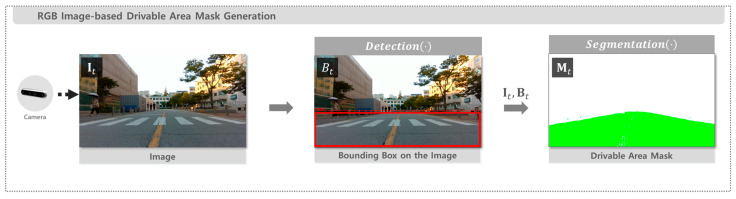
Pipeline for Drivable Area Mask Generation from RGB Image.

**Figure 3:**
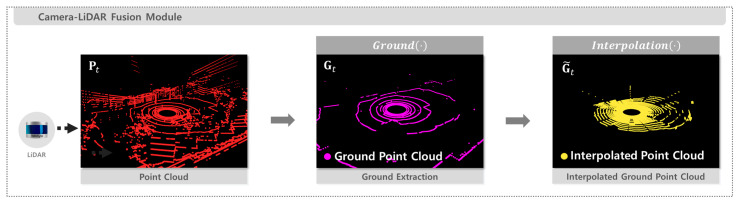
Pipeline for LiDAR-Based Ground Extraction and Interpolation.

The proposed system processes camera images and LiDAR point clouds to identify drivable areas where a robot can safely operate and generate a drivable area map. As illustrated in Figure 1, the overall architecture comprises four main stages. In Figure 1(a-1), which represents camera-based drivable area mask generation, the RGB image obtained from the camera is processed to generate drivable area masks. In Figure 1(a-2), for LiDAR-based ground point cloud generation, the point cloud obtained from LiDAR is processed to produce an interpolated ground point cloud. In Figure 1(a-3), which shows camera–LiDAR fusion, the interpolated ground point cloud is projected onto the image plane, and only the points intersecting the drivable area mask are retained to produce a high-confidence drivable area point cloud. Finally, in Figure 1b, which represents global drivable area map generation, the drivable area point cloud is transformed from the LiDAR coordinate frame into the global coordinate frame using SLAM-estimated robot poses and incrementally accumulated to construct the global drivable area map. Steps (a-1) to (a-3) collectively form the local drivable area recognition module, and step (b) corresponds to the global drivable area map generation module.

### 2.2. RGB-Image-Based Drivable Area Mask Generation

The RGB image obtained from the camera is processed to generate a drivable area mask. At each time step t, the image is denoted as $I_{t}\in R^{H\times W\times 3}$, where H and W represent the image height and width, respectively, and the last dimension corresponds to three color channels. The object detection function Detection(⋅) uses the YOLOv11s model to process $I_{t}$ as an input and detects regions corresponding to drivable areas, outputting bounding boxes $B_{t}$. The segmentation function Segmentation(⋅) then takes $I_{t}$ and $B_{t}$ as inputs to generate the final drivable area mask $M_{t}$ for detected regions. Several models have been employed for this segmentation step, including SAM 2.1 Tiny [24], FastSAM-x [25], MobileSAM [26], and YOLOv11-seg-l.

Here, $M_{t}$ denotes a binary image mask, where $M_{t}\left(u,\;v\right)=1$ indicates that pixel $\left(u,\;v\right)$ belongs to the drivable area, and a value of zero is assigned otherwise, as defined in Equation (1).

(1) $$M_{t}(u,\;v)=\left\{\begin{array}{l} 1,\;if\;pixel(u,\;v)\;belongs\;to\;a\;drivable\;area\\ 0,\;otherwise \end{array}\right.$$

The complete process of camera-based drivable area mask generation is presented in Algorithm 1, and the corresponding pipeline is illustrated in Figure 2.
**Algorithm 1:** Camera-based Drivable Area Mask Generation Process**Input:** $\;\;\;\;\;I_{t}$: RGB image at time t**Output:** $\;\;\;\;\;M_{t}$: drivable area mask at time
t1$B_{t}$ ← $Detection(I_{t})$;2$M_{t}$ ← $Segmentation(I_{t},\;B_{t})$;3$\mathrm{return}\;M_{t}$

### 2.3. LiDAR-Based Ground Point Cloud Extraction

The raw point cloud obtained from the LiDAR sensor is processed to extract and interpolate ground points. At each time step t, the point cloud is denoted as $P_{t}=\left\{p_{t,1},\ldots ,p_{t,N}\right\}$, where each point $p_{t,i}\in R^{3}$ represents a 3D coordinate in Cartesian space. The ground point extraction function Ground(⋅) separates ground and non-ground points from $P_{t}$ based on non-uniform polar coordinate partitioning and region-wise plane fitting. This operation is implemented using a patchwork algorithm [27].

The resulting ground point cloud $G_{t}$ is sparse owing to the low number of LiDAR channels. The function $Interpolation\left(\cdot \right)$, based on the spherical range image interpolation method described in [28], is applied to address this sparsity and more accurately represent the ground surface. Each ground point $g_{t,i}=(x,\;y,z)\in G_{t}$ obtained from the function Ground(⋅), is first converted into spherical coordinates as defined in Equation (2).

(2) $$r=\sqrt{x^{2}+y^{2}+z^{2}},\;\theta =atan2\left(y,x\right),\;\phi =atan2(z,\sqrt{x^{2}+y^{2}})$$

Here, r is the range, θ is the azimuth (horizontal angle), and ϕ is the elevation (vertical angle). The azimuth–elevation domain is then discretized with angular resolutions ${∆}_{\theta }$ and ${∆}_{\phi }$, and each point is mapped to a spherical range image (SRI) pixel defined by Equation (3).

(3) $$m=\left\lfloor \frac{\theta -\theta_{min}}{{∆}_{\theta }}\right\rfloor ,\;n=\left\lfloor \frac{\phi -\phi_{min}}{{∆}_{\phi }/k}\right\rfloor ,\;R\left(n,m\right)=r$$

Here, ${∆}_{\theta }$ and ${∆}_{\phi }$ denote the angular step sizes in the horizontal (azimuth) and vertical (elevation) directions, respectively, and $\left\lfloor \cdot \right\rfloor$ denotes the floor operator. The vertical step is refined by a factor ${∆}_{\phi }^{\prime }={∆}_{\phi }/k$, i.e., k, which increases the number of elevation bins by k and thus densifies the vertical sampling.

Missing values between LiDAR beams are estimated by bilinear interpolation as defined in Equation (4).

(4) $$\hat{R}\left(m,n\right)=\sum_{m^{\prime },n^{\prime }}\left(1-\left|m-m^{\prime }\right|)(1-\left|n-n^{\prime }\right|)R(n^{\prime },m^{\prime }\right)$$

Here, $m^{\prime }$, $n^{\prime }$ denote the indices of the neighboring grid points around $\left(m,n\right)$ in the SRI. In the standard bilinear setting, only the four closest neighbors contribute non-zero weights, and their contributions are summed to obtain $\hat{R}\left(m,n\right)$.

Finally, the interpolated spherical coordinates are back-projected into Cartesian space as defined in Equation (5).

(5) $$\tilde{x}=\hat{R}\left(m,n\right)cos\phi_{n}cos\theta_{m},\;\tilde{y}=\hat{R}\left(m,n\right)cos\phi_{n}cos\theta_{m},\;\tilde{z}=\hat{R}\left(m,n\right)sin\phi_{n}$$

Here, $\theta_{m}=\theta_{min}+(m-1){∆}_{\theta }$ and $\phi_{m}=\phi_{min}+(n-1)\frac{{∆}_{\phi }}{k}$.

The interpolated ground point is denoted as $\tilde{g}=(\tilde{x},\tilde{y},\tilde{z})$, and the final ground point cloud following interpolation is defined by Equation (6).

(6) $${\tilde{G}}_{t}=\left\{{\tilde{g}}_{t,1},\ldots ,{\tilde{g}}_{t,N_{g}}\right\},\;i=1,\ldots ,N_{g},\;{\tilde{g}}_{t,i}\in R^{3}$$

The complete process of LiDAR-based ground point cloud extraction is summarized in Algorithm 2, and the corresponding pipeline is illustrated in Figure 3.
**Algorithm 2:** LiDAR-based Ground Point Cloud Extraction Process**Input:** $\;\;\;\;\;P_{t}$: LiDAR point cloud at time
t**Output:** $\;\;\;\;\;{\tilde{G}}_{t}$: interpolated ground point cloud at time
t1$G_{t}$ ← $Ground(P_{t})$;2${\tilde{G}}_{t}$ ← $Interpolation(G_{t})$;3$\mathrm{return}\;{\tilde{G}}_{t}$

### 2.4. Camera–LiDAR Fusion

The drivable area mask $M_{t}$ from Equation (1) and interpolated ground point cloud ${\tilde{G}}_{t}$ from Equation (6) are used as inputs to generate a colored drivable area point cloud, retaining only the points located at the intersection of the mask and ground point cloud. Each ground point ${\tilde{g}}_{t,i}\in {\tilde{G}}_{t}$ is projected onto the image plane using the ProjectToImage(⋅) function. This function first applies the extrinsic transformation $\left[R_{ext}|t_{ext}\right]$, which represents the relative rotation and translation between the LiDAR and the camera, to convert the 3D point into the camera coordinate system. The transformed point is then multiplied by the intrinsic matrix K, which encodes the camera’s focal lengths and principal point, to map it onto the image plane in homogeneous coordinates. Finally, normalization by the depth z yields the 2D pixel coordinates $q_{t,i}=(u_{t,i},v_{t,i})$, as defined in Equation (7).

(7) $$q_{t,i}=\frac{1}{z}\cdot K\cdot \left[R_{ext}|t_{ext}\right]\cdot {\tilde{g}}_{t,i}$$

If the projected pixel coordinates $q_{t,i}$ lie inside mask $M_{t}$, the corresponding point is classified as drivable. To avoid false positives near ambiguous mask boundaries, all points projected onto the mask edges were discarded. The RGB color $c_{t,i}=\left[R_{t,i},G_{t,i},B_{t,i}\right]$ at the same pixel location in image $I_{t}$ is then assigned to that point. Through this process, a colored drivable area point cloud $D_{t}^{RGB}$ is generated, as defined in Equation (8).

(8) $$D_{t}^{RGB}=\left\{d_{t,1},\ldots ,d_{t,N_{d}(t)}\right\},\;j=1,\;\ldots ,N_{d}(t)$$

**Figure 4:**
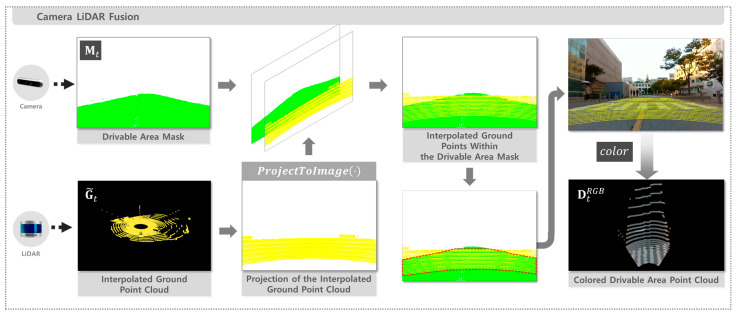
Pipeline for generating a colored drivable area point cloud via sensor fusion.

The generation of $D_{t}^{RGB}$ is summarized in Algorithm 3 and illustrated in Figure 4.
**Algorithm 3:** Camera LiDAR Fusion Process**Input:** $\;\;\;\;\;I_{t}$: RGB image at time
t $\;\;\;\;\;M_{t}$: drivable area mask at time t $\;\;\;\;\;{\tilde{G}}_{t}=\left\{{\tilde{g}}_{t,1},\ldots ,{\tilde{g}}_{t,N_{g}}\right\}$: interpolated ground point cloud at time
t**Output:** $\;\;\;\;\;D_{t}^{RGB}=\left\{d_{t,1},\ldots ,d_{t,N_{d}(t)}\right\}$: colored drivable area point cloud at time
t1  $D_{t}^{RGB}$ ← ∅;2  for i=1 $\mathrm{to}\;N_{g}$ **do**3  $q_{t,i}\leftarrow$ ProjectToImage$\left({\tilde{g}}_{t,i}\right)$;4  $\;\;\;\mathrm{if}\;M_{t}\left(q_{t,i}\right)=1$ **then**5  $\;\;\;\;\;\;c_{t,i}$ ← $I_{t}\left(q_{t,i}\right)$;6  $\;\;\;\;\;\;d_{t,i}$ ← $\left[{\tilde{g}}_{t,i}\;|\;c_{t,i}\right]$;7  $\;\;\;\;\;\;D_{t}^{RGB}$ ← $D_{t}^{RGB}\cup \left\{d_{t,i}\right\};$8     **end**9  **end**10$\mathrm{return}\;D_{t}^{RGB}$

### 2.5. Global Drivable Area Map Generation

To construct the global drivable area map $D^{global}$, the colored drivable area point clouds $D_{t}^{RGB}$, obtained from Algorithm 3, are transformed into a global coordinate frame and incrementally accumulated over time. LiDAR-based SLAM is employed to estimate the pose of the robot for each frame. Only the results of keyframes selected according to the internal criteria of the SLAM system are incorporated into the global map.

The function EstimatePoseUsingSLAM(⋅) uses the LIO-SAM [29] algorithm to estimate the robot’s global pose $T_{t}^{slam}\in R^{3\times 4}$ from $P_{t}$ at time t. Although SLAM continuously updates pose and map information, this process is simplified as a single function call in the pseudocode.

A colored drivable area point ${d_{t,j}\in D}_{t}^{RGB}$, defined in Equation (8), consists of a coordinate and color component. These components can be accessed using the functions pos(⋅) and color(⋅), which return the coordinate and RGB color of a point, respectively.

The IsKeyframe(⋅) function decides whether the current frame should be selected as a keyframe based on two criteria. A frame is selected as a keyframe when the sensor has moved more than 1 m in translation compared to the last keyframe, or when the orientation change relative to the previous frame exceeds 0.2 rad. If either of these conditions is satisfied, the current frame is designated as a keyframe and the corresponding $D_{t}^{RGB}$ is transformed into the global coordinate frame by applying the function TransformToWorld(⋅) to the coordinate component $pos(d_{t,j})$ of each point $d_{t,j}$, as defined in Equation (9).

(9) $$g_{t,j}^{world}=T_{t}^{slam}\left[\begin{array}{c} pos(d_{t,j})\\ 1 \end{array}\right],\;j=1,\;\ldots ,N_{d}(t)$$

Here, $T_{t}^{slam}=\left[R_{t}^{slam}|t_{t}^{slam}\right]\;and\;R_{t}^{slam}\in R^{3\times 3}$ is the rotation matrix, and $t_{t}^{slam}\in R^{3}$ is the translation vector. The transformed global point is denoted as $g_{t,j}^{world}$ and the color information $color(d_{t,j})$ of each point is preserved during transformation.

**Figure 5:**
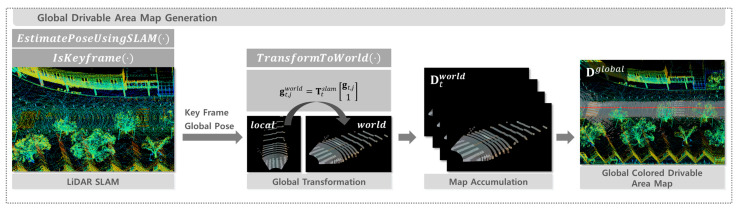
Pipeline for global colored drivable area map generation via SLAM.

Finally, the transformed colored drivable area point clouds $D_{t}^{world}$ from all keyframes are accumulated to generate the global colored drivable area map $D^{global}$. The complete procedure is summarized in Algorithm 4 and illustrated in Figure 5.
**Algorithm 4:** Global Drivable Area Map Generation Process**Input:** $\;\;\;\{P_{t}{\}}_{t=1}^{T}$: sequence of LiDAR point clouds $\;\;\;\{D_{t}^{RGB}{\}}_{t=1}^{T}$: sequence of colored drivable area point clouds, $\;\;\;\mathrm{where}\;D_{t}^{RGB}=\left\{d_{t,1},\ldots ,d_{t,N_{d}(t)}\right\}$, as defined in Equation (8)**Output:** $\;\;\;D^{global}$: global colored drivable area map1  $D^{global}$ ← ∅;2  for t=1 to T **do**3  $\;\;T_{t}^{slam}$ ← $EstimatePoseUsingSLAM\left(P_{t}\right);$4    if IsKeyframe(t) **then**5  $\;\;\;\;D_{t}^{world}$ ← ∅;6      for j=1 $\mathrm{to}\;N_{d}(t)$ **do**7  $\;\;\;\;\;\;g_{t,j}^{world}\leftarrow$ $TransformToWorld(pos(d_{t,j}),T_{t}^{slam})$;8  $\;\;\;\;\;\;d_{t,j}^{world}$ ← $\left[g_{t,j}^{world}|color(d_{t,j}\right)$]9  $\;\;\;\;\;\;D_{t}^{world}$ ← $D_{t}^{world}\cup \{d_{t,j}^{world}\};$10    **end**11$\;\;\;\;D^{global}$ ← $D^{global}\cup D_{t}^{world};$12  **end**13**end**14$\mathrm{return}\;D^{global}$

## 3. Results

### 3.1. Qualitative Evaluation on a Custom Dataset

To evaluate the effectiveness of the proposed system in a real-world environment, qualitative experiments were conducted using a custom dataset collected from a university campus. This evaluation focused on assessing drivable area recognition performance, accuracy of map visualization, and robustness of the system against both static and dynamic obstacles. Experiments were conducted under two distinct scenarios: road and sidewalk environments. All experiments were performed under identical hardware conditions using an NVIDIA RTX 3090 GPU, Intel Core i9-14900K CPU, and 32 GB of RAM on an Ubuntu 20.04 system with the robot operating system (ROS) Noetic. The movie of experimental is presented as Supplementary Material (Video).

#### 3.1.1. Sensor Configuration

To evaluate the proposed system under real-world conditions, a custom LiDAR camera–inertial measurement unit (IMU) dataset was collected using the Clearpath Jackal mobile robot platform (Clearpath Robotics, Kitchener, ON, Canada). The platform was equipped with a Velodyne VLP-16 LiDAR (Velodyne Lidar Inc., San Jose, CA, USA) for ground point extraction, interpolation, and SLAM-based global drivable area map generation. An Intel RealSense D435 RGB-D camera (Intel Corporation, Santa Clara, CA, USA) was used for drivable area mask generation from RGB images. A VectorNav VN-100 IMU (VectorNav Technologies, Dallas, TX, USA) was used as an auxiliary sensor to improve pose estimation and maintain SLAM consistency. All sensors were synchronized in real time using ROS (ROS, version Noetic, Open Source Robotics Foundation, Mountain View, CA, USA), enabling accurate data fusion and robust operation of the proposed pipeline. The camera and LiDAR were rigidly mounted with their optical axes aligned, with the camera positioned 14.3 cm in front of the LiDAR and 14.9 cm below it. The robot dimensions are 43 cm in width and 50.8 cm in length.

**Figure 6:**
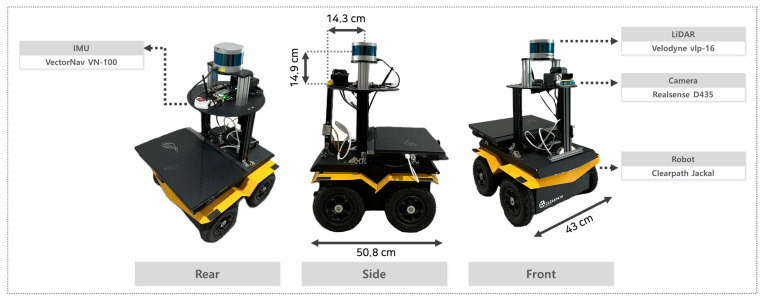
Sensor configuration used in our experiments.

The overall sensor configuration of the mobile robot is presented in Figure 6.

#### 3.1.2. Recognition and Map Generation of Drivable Areas in a Road Environment

**Figure 7:**
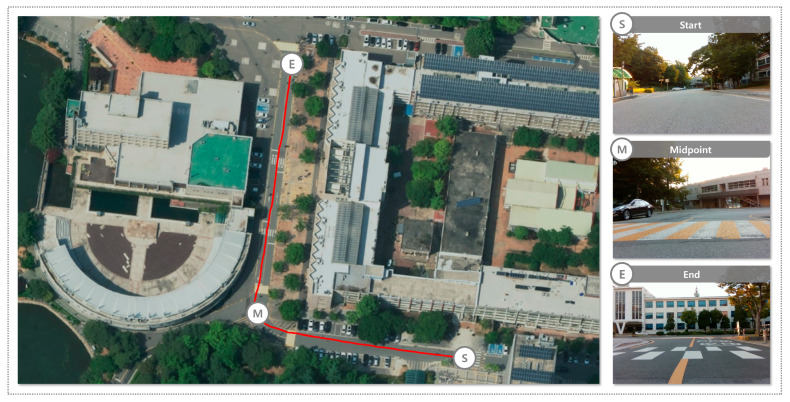
Custom dataset route in a road environment.

To evaluate the performance of the proposed system in a road environment, the data collection route is presented in Figure 7. The route spans approximately 200 m and includes various road geometries and surrounding conditions to reflect diverse scenarios that may appear during real-world driving. The road dataset was collected at dusk under clear weather conditions, providing natural illumination changes caused by low-angle sunlight. The route included both straight and curved segments as well as multiple road features such as lane markings, speed bumps, and pedestrian crosswalks. These elements were selected to replicate realistic urban driving conditions and to examine the robustness of the proposed system across different road structures and traffic-related features.

**Figure 8:**
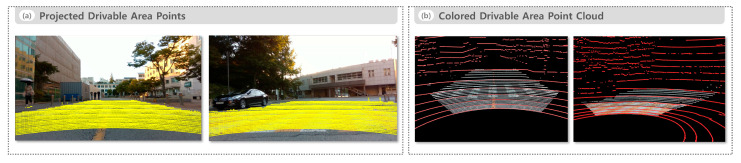
Drivable area recognition results in the road environment. (**a**) Projected drivable area points overlaid on a camera image and (**b**) colored drivable area point cloud overlaid on the raw LiDAR point cloud.

Figure 8 presents the drivable area recognition results for the road environment. Figure 8a illustrates the projected drivable area points overlaid on the camera image, clearly identifying actual drivable regions while effectively excluding road boundaries and non-drivable areas. Figure 8b presents the colored drivable area point cloud overlaid on the raw LiDAR point cloud, providing an intuitive representation of the shape and spatial distribution of drivable regions in 3D space.

**Figure 9:**
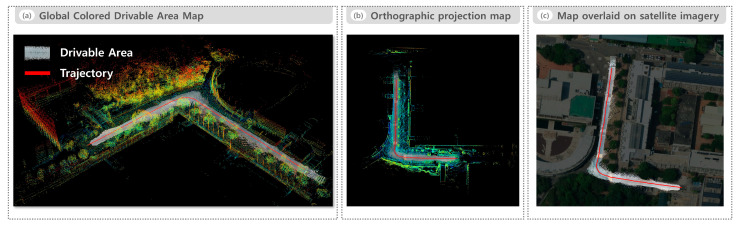
Visualization of the global colored drivable area map in the road environment: (**a**) Global colored drivable area map in 3D overlaid on the raw LiDAR point cloud map, (**b**) orthographic projection map obtained by projecting both the global colored drivable area map and raw LiDAR point cloud map onto a 2D plane, and (**c**) 2D drivable area map obtained from the orthographic projection in (**b**) overlaid on high-resolution satellite imagery.

Figure 9 presents the visualization results of the global colored drivable area map generated for the road environment. Figure 9a depicts the global colored drivable area map in 3D space overlaid on the raw LiDAR point cloud map generated through SLAM-based accumulation. Recognized drivable regions are continuously and uniformly connected along the robot’s trajectory, even in areas with curved road sections or complex lane structures. Figure 9c overlays the orthographically projected 2D drivable area map onto high-resolution satellite imagery. For this alignment, four GPS-referenced landmarks (e.g., road intersections and building corners) located outside the drivable area were manually selected, and a homography transformation was estimated to align the satellite image with the map coordinate frame. Placing the landmarks outside the evaluation region prevents extrapolation artifacts within the drivable area itself. The resulting overlay demonstrates high geometric consistency between the recognized drivable areas and the actual road layout.

#### 3.1.3. Recognition and Map Generation of Drivable Areas in a Sidewalk Environment

**Figure 10:**
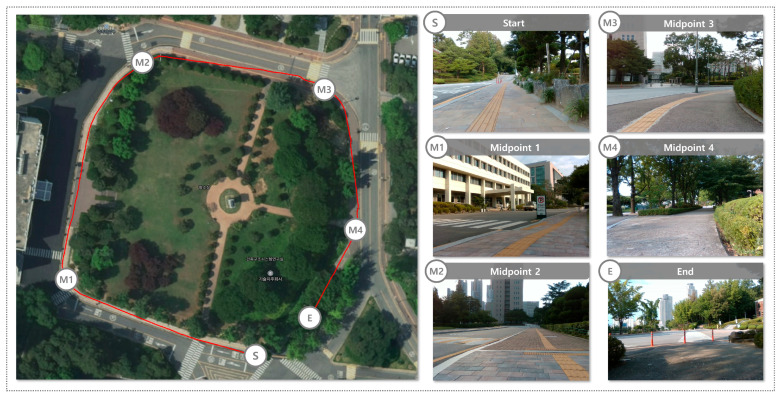
Custom dataset route in a sidewalk environment.

To evaluate the performance of the proposed system in a sidewalk environment, the data collection route is presented in Figure 10. The route spans approximately 350 m and includes various pedestrian-related features such as narrow walkways, patterned pavement blocks, street trees, streetlights, and temporary structures. These conditions were selected to reflect diverse navigation constraints specific to sidewalk environments, which differ from those in road environments. The dataset was collected during the daytime under clear weather conditions. The route contained both straight and curved sidewalk segments, and was surrounded by dense vegetation such as street trees and shrubs, providing frequent occlusions and irregular boundaries. These environmental factors introduced additional complexity to drivable area recognition and allowed us to assess the robustness of the system in realistic pedestrian spaces.

**Figure 11:**
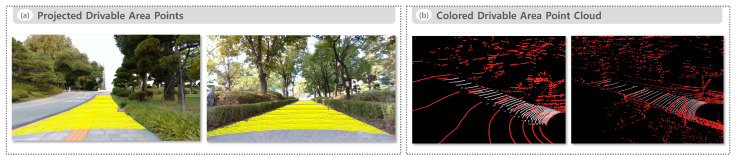
Drivable area recognition results in the sidewalk environment: (**a**) projected drivable area points overlaid on a camera image and (**b**) colored drivable area point cloud overlaid on a raw LiDAR point cloud.

Figure 11 presents the drivable area recognition results for the sidewalk environment. Figure 11a illustrates the projected drivable area points overlaid on a camera image, effectively excluding non-drivable regions such as curbs and grass strips, while clearly identifying actual drivable areas. Figure 11b presents the colored drivable area point cloud overlaid on the raw LiDAR point cloud, providing an intuitive representation of the spatial extent and distribution of drivable areas in 3D space under various environmental conditions.

**Figure 12:**
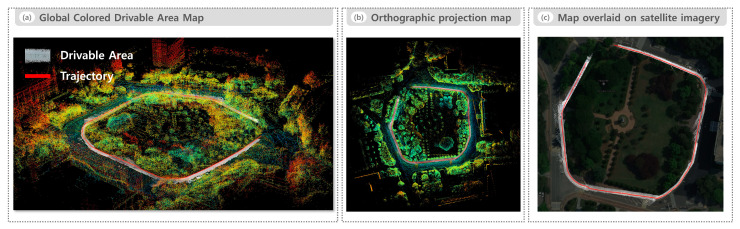
Visualization of the global colored drivable area map in the sidewalk environment: (**a**) global colored drivable area map in 3D overlaid on the raw LiDAR point cloud map, (**b**) orthographic projection map obtained by projecting both the global colored drivable area map and raw LiDAR point cloud map onto a 2D plane, and (**c**) 2D drivable area map obtained from the orthographic projection in (**b**) overlaid on high-resolution satellite imagery.

Figure 12 presents the visualization results of the global colored drivable area map generated in the sidewalk environment. Figure 12a depicts the global colored drivable area map in 3D space overlaid on the raw LiDAR point cloud map, generated through SLAM-based accumulation. The recognized drivable regions are continuously and uniformly connected along the robot trajectory, even in areas with irregular pavement patterns or narrow paths. Figure 12b presents the orthographic projection map obtained by projecting both the global colored drivable area map and raw LiDAR point cloud map onto a 2D plane. Figure 12c overlays the orthographically projected 2D drivable area map onto high-resolution satellite imagery, aligned using the same landmark-based homography method described in Figure 9c. The resulting overlay demonstrates high geometric consistency between the recognized drivable areas and the actual sidewalk layout.

#### 3.1.4. Obstacle-Aware Drivable Area Recognition

To ensure safe and reliable navigation in real-world scenarios, drivable area recognition must accurately exclude both static and dynamic obstacles present in the environment. These obstacles can interfere with path planning and potentially lead to unsafe maneuvers if they are not properly identified. In this study, we conducted a qualitative visual evaluation of the system’s ability to recognize drivable areas while excluding both static and dynamic obstacles.

**Figure 13:**
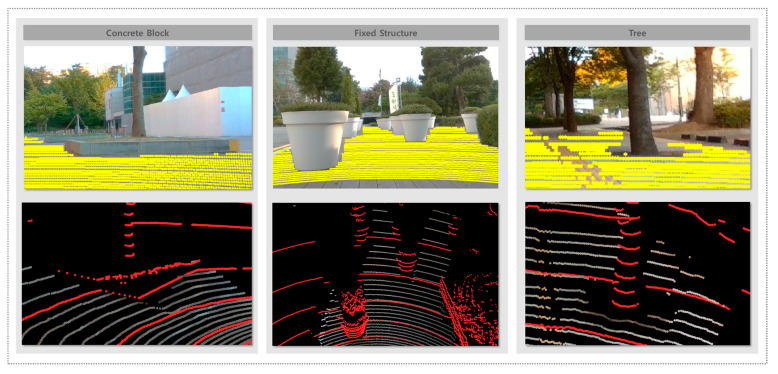
Drivable area recognition in the presence of static obstacles.

Figure 13 presents the system response to static obstacles. In each example, projected drivable points and colored drivable area point clouds clearly demonstrate that the system successfully recognizes drivable areas while avoiding regions with concrete blocks, trees, and other fixed structures. These results confirm that the system is capable of identifying and excluding non-traversable areas, even when they are stationary and structurally variable.

**Figure 14:**
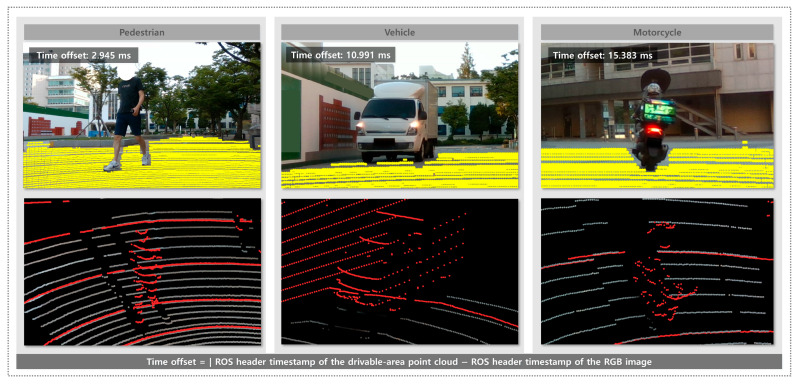
Drivable area recognition in the presence of dynamic obstacles. Each example is annotated with the time offset between the RGB image and the drivable area point cloud, computed from ROS header timestamps.

Figure 14 presents the response of the system to dynamic obstacles. In each example, the projected drivable points and 3D RGB drivable area point cloud clearly demonstrate that the system successfully identified drivable areas while excluding the regions occupied by pedestrians, vehicles, and motorcycles. These results confirm that the system can effectively adapt to moving obstacles and maintain safe drivable area recognition in dynamic environments. For transparency, each example is annotated with the time offset between the RGB image and the drivable area point cloud, computed from ROS header timestamps. The absolute time offsets are 2.945 ms, 10.991 ms, and 15.383 ms for the pedestrian, motorcycle, and car example, respectively.

#### 3.1.5. Failure Cases

**Figure 15:**
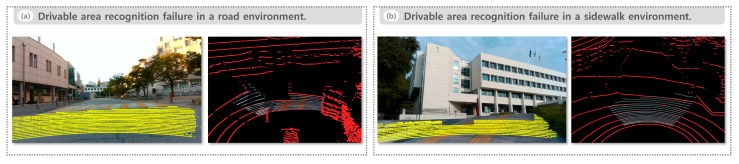
Failure cases of drivable area recognition. (**a**) Drivable area recognition failure in a road environment, and (**b**) drivable area recognition failure in a sidewalk environment.

We include representative failure cases to clarify the boundary conditions of the proposed pipeline. Figure 15a presents a failure in a road environment with a pronounced speed bump. The method does not recognize the bump as drivable, and the drivable area point cloud becomes locally conservative. Figure 15b presents a failure in a sidewalk environment on a tight curve. The predicted drivable region extends beyond the sidewalk, crossing the curb line into the adjacent roadway on the left and resulting in a false-positive extension.

#### 3.1.6. Effect of Interpolation on Global Drivable Area Map Continuity

**Figure 16:**
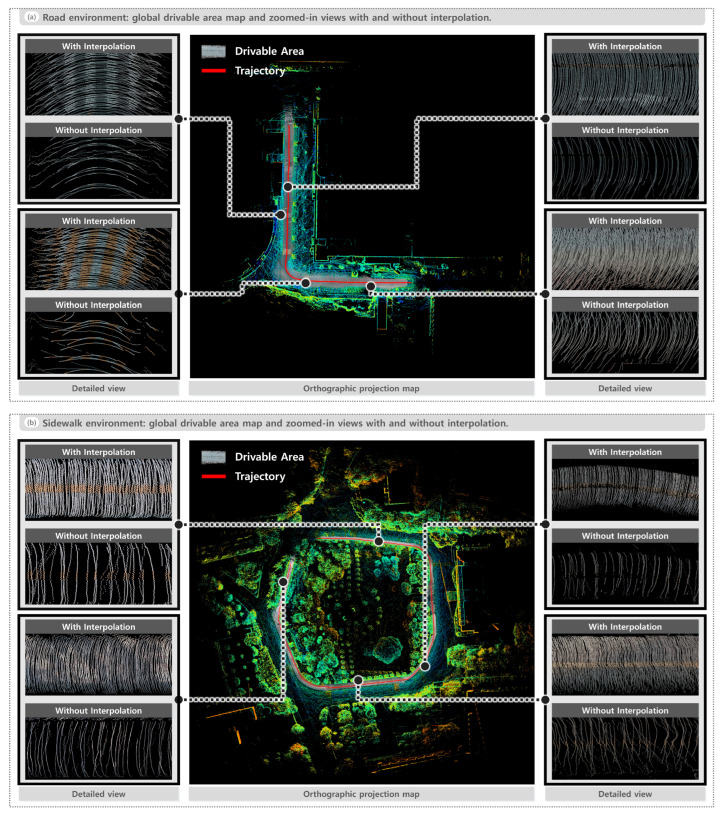
Comparison of global drivable area maps with and without interpolation. (**a**) Road environment. (**b**) Sidewalk environment. Each includes a global drivable area map and zoomed-in views highlighting the difference between interpolation and non-interpolation results.

To investigate the effect of interpolation on global drivable area map generation, we produced maps both with and without applying the interpolation step. As shown in Figure 16, the interpolated results provide denser ground representations and smoother continuity, making fine-grained surface features such as speed bumps and crosswalks more visible. In contrast, the non-interpolated maps appear sparser, with discontinuities along drivable area boundaries and less clearly defined ground details.

### 3.2. Quantitative Evaluation on the KITTI Dataset

To evaluate the accuracy and efficiency of the proposed drivable area recognition system, we conducted quantitative experiments using the SemanticKITTI [10] dataset, which contains large-scale urban driving scenes with point-level semantic annotations.

#### 3.2.1. Evaluation Setup

We evaluated the performance of the proposed drivable area recognition method through comparative experiments with existing LiDAR-based semantic segmentation algorithms. Baseline models [15,16,17,18,19] were trained on sequences 00 to 07, 09, and 10 in the SemanticKITTI dataset [10], following the standard evaluation protocol where sequence 08 is reserved exclusively for validation. Our evaluation adhered to this protocol using sequence 08 to ensure fair comparison and reproducibility.

Because the proposed system recognizes drivable areas by fusing RGB-based segmentation with LiDAR-derived ground points, the evaluation was restricted to the actual FOV of the camera, where RGB perception is applicable. For each frame, the LiDAR points were transformed into a camera coordinate frame and projected onto an image plane. Only points within the KITTI image resolution (375 × 1241 pixels) were considered, ensuring consistency between the evaluation region and the effective range of sensor fusion.

This quantitative evaluation focused on a road class with a class ID of 40 in the SemanticKITTI [10] label set. This is because we define the drivable area as a continuous planar surface that the robot is currently navigating, and in the SemanticKITTI [10] dataset, the vehicle consistently drives on roads. Therefore, the road class best represents the drivable area within this dataset. The ground-truth labels from SemanticKITTI [10] served as references for comparison. Although our pipeline includes an interpolation module to densify sparse ground points, this module was excluded from the evaluation because interpolation can extend predictions into regions without ground-truth labels, potentially introducing bias. Therefore, the evaluation was limited to points with available ground-truth labels, enabling a direct and fair assessment.

#### 3.2.2. Evaluation Setup and Metrics

The proposed system was quantitatively evaluated using two commonly adopted metrics for road classes: intersection over union (IoU) and precision. IoU is defined in Equation (10), and precision is defined in Equation (11).

(10) $${IoU}_{road}=\frac{TP}{TP+FP+FN}$$

(11) $${Precision}_{road}=\frac{TP}{TP+FP}$$

- True Positive (TP): Number of LiDAR points correctly predicted as roads.
- False Positive (FP): Number of points predicted as roads but actually belonging to non-road areas.
- FN (False Negative): Number of road points incorrectly predicted as non-roads.

Equation (10) measures the degree of overlap between the predicted road region and ground-truth labels, reflecting overall segmentation accuracy. Equation (11) calculates the proportion of correctly predicted road points among all points classified as roads. This measure is particularly useful for evaluating the effectiveness of a method at reducing false positives.

Evaluation was conducted in a frame-by-frame manner. IoU and precision values were computed for each frame, and the average values were used to compare performances across different algorithms. Frames per second (FPS) was measured in an end-to-end manner, starting from the input of sensor data and continuing until the final output of drivable area point clouds. For our method, this includes RGB segmentation, LiDAR ground segmentation, and fusion, while for LiDAR-only baselines it corresponds to the complete model inference pipeline. All methods were executed on the same hardware platform described earlier to ensure a fair comparison.

#### 3.2.3. Performance Comparisons with LiDAR-Based Segmentation Models

The performance of the proposed drivable area recognition method was compared with that of representative LiDAR-based semantic segmentation algorithms, including 2DPASS [19], MinkowskiNet [18], Cylinder3D [17], SPVCNN [15], and RPVNet [16]. All experiments were conducted under identical conditions, following the aforementioned evaluation setup, which measured the IoU, precision, and FPS for the road class (ID 40) within the FOV of the camera.

**Figure 17:**
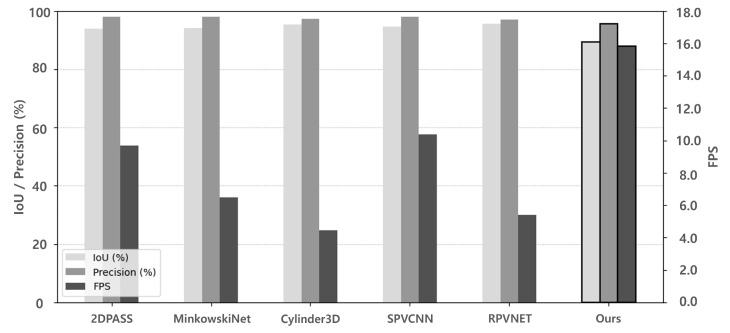
Performance graph of the proposed and LiDAR-based segmentation models including 2DPASS [19], MinkowskiNet [18], Cylinder3D [11], SPVCNN [16], RPVNET [17]. All FPS values were measured on the same hardware platform (RTX 3090 GPU, Intel Core i9-14900K CPU) to ensure consistent comparison.

Table 1 summarizes the comparison results while Figure 17 visualizes the results. Existing LiDAR-based models achieved high IoU (≥93.79%) and precision (≥96.94%) but operated at lower speeds, with FPS values ranging from 4.46 to 10.37. In contrast, the proposed method recorded a slightly lower IoU of 89.34% and precision of 95.62% but delivered the highest inference speed of 15.82 FPS. This result demonstrates the real-time performance of the proposed method, making it suitable for applications that require fast processing while maintaining competitive accuracy.

### 3.3. RGB-Based Segmentation Module Comparison

To identify the most suitable RGB-based segmentation module for the proposed drivable area recognition system, we evaluated the effects of different segmentation models on overall system performance. Because this module is responsible for generating drivable area masks used in sensor fusion, its accuracy directly impacts recognition performance, and its speed influences real-time operation feasibility. Therefore, selecting an optimal model is essential for achieving both high segmentation quality and efficiency. A set of controlled experiments were conducted, where the overall pipeline was fixed and only the segmentation model was replaced with one of four candidates: SAM 2.1 Tiny [24], FastSAM-x [25], MobileSAM [26], and YOLOv11-seg-l.

All experiments were conducted under identical conditions using sequence 08 of the SemanticKITTI [10] dataset, following the evaluation setup described previously. The evaluation was restricted to the camera’s FOV and focused exclusively on the road class (ID 40). The same performance metrics described previously were used again.

**Figure 18:**
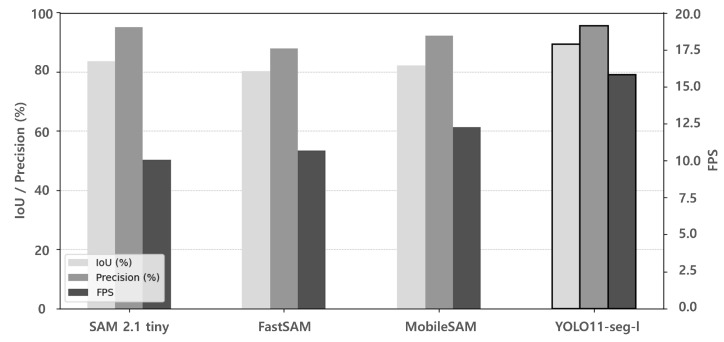
Performance graph of RGB segmentation modules including SAM2.1 tiny [24], FastSAM [25], MobileSAM [26], and YOLO11-seg-l.

Table 2 summarizes the results of the comparative evaluations. Among the candidates, YOLOv11-seg-l achieved the highest IoU (89.34) and FPS (15.82), while maintaining competitive precision. These results demonstrate its effectiveness in terms of both segmentation accuracy and real-time performance, making it the optimal choice for integration into the proposed system. The same results are visualized in Figure 18, which compares the performance of each model in terms of IoU, precision, and FPS.

In addition to segmentation modules, we also compared several YOLO variants (YOLOv7, YOLOv8s, YOLOv9s, YOLOv11s, and YOLOv12s) as the object detection component. While the performance differences in terms of IoU and FPS were relatively small, YOLOv11s achieved the best balance between accuracy and inference speed. Therefore, YOLOv11s was adopted as the final object detection model in our system.

### 3.4. Evaluation Under Different LiDAR Channel Configurations

To further examine the robustness of the proposed framework across different LiDAR configurations, we evaluated the system under varying channel counts (64, 32, 16, and 8). We followed the same evaluation protocol as in the previous experiments, restricting the evaluation to the camera’s FOV and the road class (ID 40) in SemanticKITTI [10]. Importantly, all experiments were conducted without any retraining or modification of the model, demonstrating that the proposed framework can directly operate across different LiDAR channel counts.

**Figure 19:**
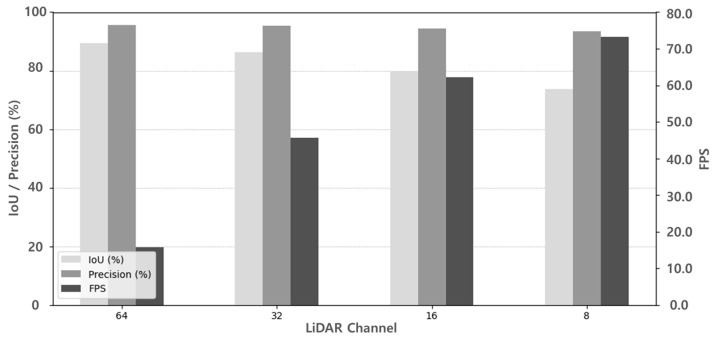
Performance graph under different LiDAR channel configurations.

As summarized in Table 3 and visualized in Figure 19, reducing the LiDAR channel count inevitably led to lower IoU and precision owing to sparser point clouds. Specifically, when the resolution was reduced from 64 to 8 channels, IoU decreased by 17.53% and precision decreased by 2.15%. These results show that the proposed framework can be applied to different LiDAR configurations without retraining.

### 3.5. Map Traversability Evaluation

To assess the usability of the generated maps for navigation, we performed a traversability analysis focusing on the available width of the drivable areas.

For this evaluation, we utilized the keyframes extracted by the IsKeyframe(⋅) function in Algorithm 4 as reference poses. At each keyframe location, we examined the distribution of points in the lateral direction relative to the robot’s heading in the local coordinate system, thereby measuring the traversable width of the drivable corridor. This procedure allowed us to quantify the passage size in both road and sidewalk environments.

**Figure 20:**
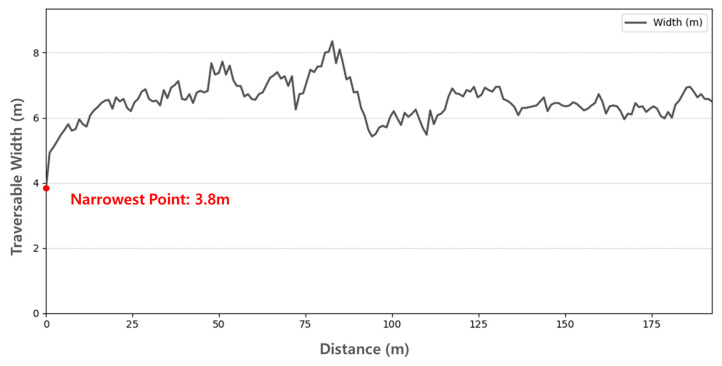
Traversable width analysis of the global drivable area map in the road environment.

**Figure 21:**
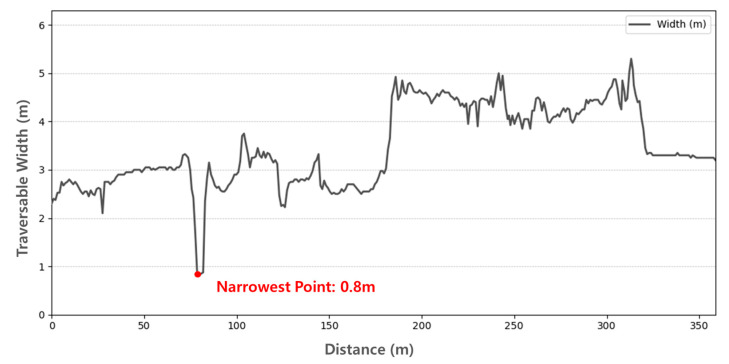
Traversable width analysis of the global drivable area map in the sidewalk environment.

As shown in Figure 20, the narrowest traversable width in the road map was measured as 3.8 m, while Figure 21 shows that the sidewalk map had the narrowest traversable width of 0.8 m. Considering the Clearpath Jackal platform has an overall width of 0.43 m as specified in the Sensor Configuration, these values indicate that the robot can safely traverse both environments, leaving safety margins of 3.37 m for the road and 0.37 m for the sidewalk, respectively.

This analysis confirms that the proposed maps are not only geometrically accurate but also practically traversable, thereby supporting their usability for stable path generation.

## 4. Discussion

Herein, we proposed a drivable area recognition system that fuses an RGB camera and LiDAR sensor. The proposed system is designed to be robust to domain differences between sensors and is applicable to diverse environments without requiring retraining. The system adopts a sensor-independent architecture capable of processing inputs regardless of the sensor type or resolution, and is implemented using an ROS framework, enabling easy integration into real-world robotic platforms. Furthermore, the system is modular in design, allowing easy replacement of individual components such as the RGB segmentation model or LiDAR preprocessing algorithm. This modularity offers flexibility and scalability, enabling future improvements in overall system performance through simple model upgrades.

In quantitative evaluations, while existing LiDAR-based semantic segmentation models achieved high accuracy with IoU scores ranging from approximately 94% to 95%, the proposed system achieved a slightly lower IoU of 89%. In terms of inference speed (FPS), which reflects real-time capabilities, the proposed system reached 15.82 FPS, significantly outperforming existing models that ranged between 4 and 10 FPS. This balance highlights the suitability of the method for real-time deployment in mobile robotics and autonomous driving. The lower IoU can be partly explained by the evaluation setting in which LiDAR-only baselines were trained on SemanticKITTI [10] sequences 00–07 and 09–10 and evaluated on sequence 08, where the similarity between training and test environments may have led to dataset-specific overfitting and inflated performance. Our pipeline, by contrast, does not depend on such dataset-specific training but fuses RGB segmentation and LiDAR ground extraction in a modular manner, enabling robustness to changes in sensors and operating conditions.

This modularity not only enhances adaptability but also facilitates performance improvements through simple component upgrades. As demonstrated in Table 2, simply replacing the RGB segmentation module with a more advanced model, such as YOLOv11-seg-l, improved IoU and speed compared to earlier models like SAM 2.1 Tiny, FastSAM-x, and MobileSAM. This demonstrates that the overall framework can be strengthened without structural changes by updating individual modules. For the RGB module, although our system already employs a foundation model–based segmentation approach, it can readily incorporate future foundation models that offer higher accuracy or stronger real-time performance, as well as more advanced transformer-based segmentation models as they are developed. For the LiDAR module, the same modular principle allows the substitution of more advanced and efficient ground segmentation algorithms, together with interpolation strategies that are both more real-time and better at preserving fine-grained structural features of the scene. In this perspective, the proposed system is not limited by its current configuration but rather provides a scalable platform that can continuously benefit from progress in RGB segmentation, LiDAR ground segmentation, and interpolation techniques.

We also examined the influence of LiDAR channel reduction to assess the framework’s generalizability. When the resolution was reduced from 64 to 8 channels, IoU decreased by 17.53% and precision decreased by 2.15%, even though no retraining was applied. These results suggest that the proposed system can still be applied across different LiDAR configurations without the need for retraining, while acknowledging that performance degradation is inevitable under extremely sparse settings.

The system demonstrated strong performance in terms of recognizing drivable areas while effectively filtering out both static and dynamic obstacles. As shown in Figure 13, the system successfully excluded various static obstacles such as concrete blocks, raised curbs, and trees from recognized drivable areas. These results indicate that the system can maintain accurate drivable area segmentation even in environments with complex and irregular fixed structures. Figure 14 illustrates the ability of the system to handle dynamic obstacles. In environments with moving objects such as pedestrians, vehicles, and motorcycles, the system reliably excludes these regions from the drivable area. These results confirm that the proposed method not only operates efficiently in static scenarios but also maintains safe drivable area recognition under dynamic conditions, which is essential for real-world deployment.

However, despite these promising results, certain limitations were observed in complex geometric configurations. Figure 15a shows a road environment where the drivable area is not recognized over a speed bump. Although this region should be considered drivable, the surface discontinuity leads to an overly conservative result. Figure 15b presents a curved sidewalk environment where the predicted drivable area incorrectly extends into the adjacent road. This misclassification likely arises from the lack of a distinct boundary or strong curvature. These examples highlight the system’s sensitivity to local geometric variations, such as bumps and curb edges, which can affect segmentation accuracy and boundary alignment.

As illustrated in Figure 9a and Figure 12a, the drivable areas recognized by the proposed system were accumulated into globally consistent maps, showing smooth connectivity and spatial coherence throughout the entire trajectory in both road and sidewalk environments. Figure 9c and Figure 12c further validate this spatial consistency by overlaying the generated maps onto high-resolution satellite imagery, clearly demonstrating their precise geometric alignment with actual road and sidewalk structures. These qualitative results confirm that the proposed fusion approach effectively generates accurate and coherent global drivable maps.

While dynamic objects are excluded from the drivable mask on a per-frame basis, the global map accumulation currently does not incorporate occupancy lifetimes or temporal consistency checks. As a result, ghost-free space could still occur when dynamic obstacles appear and disappear across consecutive frames. In practice, such empty regions are often filled by subsequent frames as the map continues to be accumulated, but the lack of explicit temporal filtering remains a limitation. This highlights the need for future extensions of the system that integrate temporal consistency mechanisms to further improve robustness in highly dynamic environments.

As illustrated in Figure 16, the comparison emphasizes the importance of interpolation in providing a clearer representation of ground features. While the overall recognition pipeline does not depend on this step, interpolation contributes to generating more continuous and visually coherent global drivable area maps, thereby improving the interpretability of the results.

Although the proposed system exhibits promising results in various structured environments, it has several limitations. The performance of the system is influenced by the accuracy of sensor calibration, particularly when aligning LiDAR point clouds with RGB images. Inaccurate extrinsic parameters can lead to misaligned projections, which may affect recognition quality in certain scenarios. To further quantify this effect, we conducted an additional experiment in which the extrinsic parameters were intentionally perturbed. Specifically, yaw rotations of 5°, 10°, and 15° were introduced to simulate miscalibration. The results showed that IoU decreased by 25.3%, 46.7%, and 62.3% at 5°, 10°, and 15° miscalibrations, respectively, while precision decreased by 11.5%, 23.3%, and 34.3%. These findings demonstrate that even small angular miscalibrations can substantially degrade recognition performance, underscoring the importance of accurate initial calibration. Nevertheless, once an accurate initial calibration is established, our system performs projection on a frame-by-frame basis, so cumulative errors do not occur, ensuring stable operation in practice.

Additionally, the current evaluation was conducted in structured environments such as roads and sidewalks, and the robustness of the system in unstructured or complex terrains such as construction sites or uneven natural surfaces has not yet been validated. The system also depends on RGB-based segmentation in its front-end processing, meaning its overall performance can be affected by the quality of the segmentation model, especially under challenging visual conditions such as shadows, reflections, or poor lighting. These aspects present important directions for future improvements and generalization. In particular, conducting systematic evaluations under adverse conditions such as nighttime scenes, glare, rain, or shadowed environments, and constructing a failure taxonomy of segmentation outcomes, would represent valuable extensions of this work. However, such efforts require datasets that provide synchronized RGB and LiDAR inputs along with pixel-level ground-truth annotations under various stress conditions. These types of resources are currently scarce. As future work, we intend to develop custom data collection strategies and evaluation protocols to address this limitation.

## 5. Conclusions

This study presented a sensor-fusion-based drivable area recognition system that integrates RGB and LiDAR data in a modular and sensor-agnostic architecture. The proposed system operates in real time without the need for retraining and is implemented using ROS, enabling seamless deployment on robotic platforms. It demonstrated robust performance in structured environments such as roads and sidewalks, effectively recognizing drivable areas. The system also succeeded in constructing globally consistent drivable maps by combining SLAM-based alignment, LiDAR ground extraction, and image-based segmentation. Furthermore, it preserved fine-grained surface features such as speed bumps and crosswalks, suggesting potential applications in semantic mapping and infrastructure-level road analysis. Notably, the modular architecture of the system allows for easy replacement and upgrading of individual components, such as segmentation or ground-filtering modules, offering flexibility for future enhancements. Although the proposed system exhibits strong practical performance, it remains sensitive to sensor calibration and has not yet been validated in unstructured or off-road environments. Future work may extend this framework to construction sites, rural paths, and other complex terrains, further broadening its applicability to real-world navigation tasks.

## Figures and Tables

**Table 1:** Performance Comparison of the Proposed Method and LiDAR-based Segmentation Algorithms on KITTI Sequence 08. All FPS values were measured on the same hardware platform (RTX 3090 GPU, Intel Core i9-14900K CPU) to ensure consistent comparison.

Methods	IoU (%)	Precision	FPS
2DPASS [19]	93.79	98.07	9.68
MinkowskiNet [18]	94.14	97.91	6.49
Cylinder3D [11]	95.22	97.28	4.46
SPVCNN [16]	94.70	97.89	10.37
RPVNET [17]	95.57	96.94	5.4
Ours	89.34	95.62	15.82

**Table 2:** Performance Comparison according to RGB segmentation modules.

Methods	IoU(%)	Precision	FPS
SAM 2.1 tiny [24]	83.52	95.12	10.06
FastSAM-x [25]	80.12	87.94	10.69
MobileSAM [26]	82.20	92.18	12.28
YOLOv11-seg-l	89.34	95.62	15.82

**Table 3:** Performance under different LiDAR channel configurations.

LiDAR Channel	IoU(%)	Precision	FPS
64	89.34	95.62	15.82
32	86.37	95.45	45.59
16	79.73	94.32	62.25
8	73.65	93.57	73.19

## Data Availability

The original contributions presented in this study are included in the article/Supplementary Materials. Further inquiries can be directed to the corresponding author.

## Supplementary Materials

The following supporting information can be downloaded at: https://www.mdpi.com/article/10.3390/s25185841/s1.

## Abbreviations

The following abbreviations are used in this manuscript:

IoU	Intersection over Union
FPS	Frames per Second
